# Supplementary material for: Effects of Dimerization, Dendrimerization, and Chirality in p-BthTX-I Peptide Analogs on the Antibacterial Activity and Enzymatic Inhibition of the SARS-CoV-2 PLpro Protein
Source: Pharmaceutics. 2023 Jan 28;15(2):436. doi: 10.3390/pharmaceutics15020436 (PMC9964244; doi:10.3390/pharmaceutics15020436)
Supplement: Supplementary file 1 [file pharmaceutics-15-00436-s001.zip › pharmaceutics-2144411-supplementary.pdf]

pharmaceutics

Supplementary Materials: Effects of Dimerization, Dendrimerization, and Chirality in p-BthTX-I Peptide Analogs on the Antibacterial Activity and Enzymatic Inhibition of the SARS-CoV-2 PL^{pro} Protein

Natália Vitória Bitencourt, Gabriela Marinho Righetto, Ilana Lopes Baratella Cunha Camargo, Mariana Ortiz de Godoy, Rafael Victorio Carvalho Guido, Glaucius Oliva, Norival Alves Santos-Filho and Eduardo Maffud Cilli

Synthesis and characterization of the peptides

The peptides were manually synthesized by solid-phase using the Fmoc (9-fluorenylmethyloxycarbonyl) protocol. The synthesis was performed using Rink Amide resin (0.55 mmol g-1, AAPPTEC) to obtain the peptide in alpha-carboxyamide form after the cleavage step. Briefly, deprotection of the Fmoc group was performed in 20% 4-methylpiperidine in dimethylformamide (DMF) by 1 and 20 min. Fmoc-amino acids were coupled using diisopropylcarbodiimide (DIC)/N-hydroxybenzotriazole (HOBt) in DMF for 2 h stirring, with 2-fold excess for all coupling reagents. If necessary, O-(benzotriazol-1-yl)-N,N,N′,N′-tetramethyluronium hexafluorophosphate (HBTU)/diisopropylethylamine (DIEA) in DMF was used. The dimer and tetramer analogs (L and D isomers) were obtained using the previously described strategy [1] using one (for dimers) or 3 (for tetramers) Fmoc-Lys(Fmoc)-OH in the C-terminal region. The chains of peptides were simultaneously elongated grown up in α- and ε-amino groups, obtaining homo dimers and tetramers. The cleavage of the peptide from the resin was performed for 2 h using 95% TFA, 2.5 TIS (triisopropylsilane) and 2.5 water, at a ratio of 10 mL/g resin. The crude peptides were precipitated with chilled ethyl ether, separated from soluble nonpeptide material by centrifugation, and lyophilized. Purification of the synthetic peptides was performed in a semi-preparative mode using a C18 reverse-phase column (AAPPTEC 1.0 × 25 cm). The purity was determined on a C18 reverse-phase (0.46 × 25 cm) analytical column (Agilent, Santa Clara, CA, USA). The solvents were 0.045% TFA in deionized water and 0.036% TFA in acetonitrile with 1 mL/min with a linear gradient of 5–95% solvent B (0.036% (v/v) TFA/acetonitrile) for 30 min. The chromatograms and mass spectra of the peptides are showed in Figure S1-S6. The mass spectrometry which indicated the molecular mass of the cation/charge ratio of the identified peptides' analysis was carried out. The mass spectra of these peptides were evaluated, and the synthesis was confirmed (Table S1).

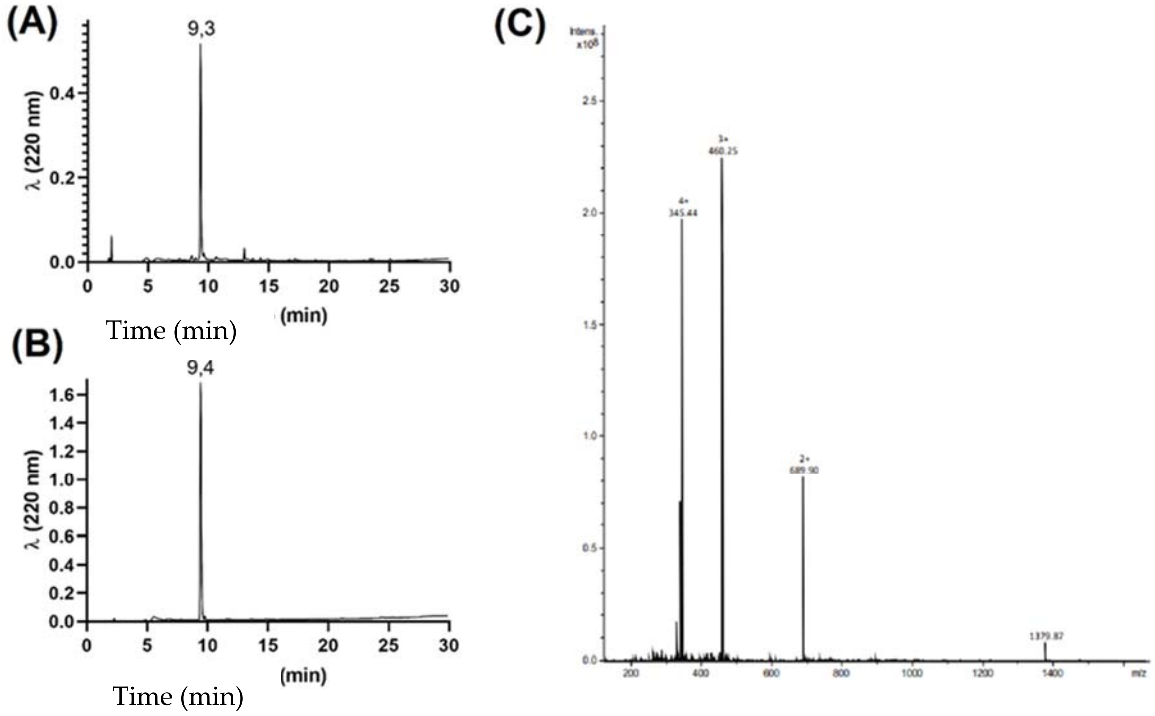

Figure S1. Chromatogram of the crude (a) and purified KKYRYHLKPF (L-monomer) peptide (b) - analytical reverse phase Phenomenex Jupiter C18 column (150 x 4.6 mm), packed with spherical 5 mm particle and 300 Å pore size, using a linear gradient of 5–95% (v/v) of solvent B for 30 min, at a flow rate of 1.0 mL/min and UV detection at 220 nm. Mass spectrum of the peptide (c) - Thermo LCQ-fleet mass spectrometer, with ESI-IT-MS configuration. Direct infusion of the sample solutions was carried out at a concentration of about 10 ppm in acetonitrile/water containing 0.1% v/v formic acid. The infusion flow was adjusted to 5.0 μL/min and the electrospray source was operated in positive mode, applying 4.5 kV to the electrospray capillary.

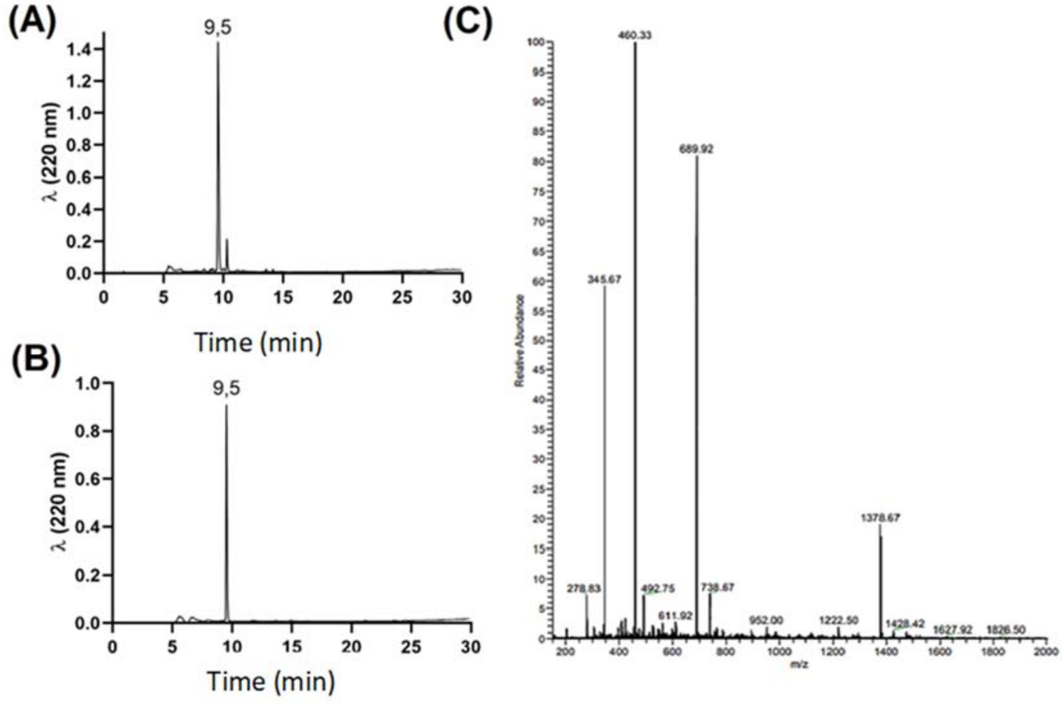

Figure S2. Chromatogram of the crude (a) and purified kkyryhlkpf (D-monomer) peptide (b) - analytical reverse phase Phenomenex Jupiter C18 column (150 x 4.6 mm), packed with spherical 5 mm

particle and 300 Å pore size, using a linear gradient of 5–95% (v/v) of solvent B for 30 min, at a flow rate of 1.0 mL/min and UV detection at 220 nm. Mass spectrum of the peptide (c) - Thermo LCQ-fleet mass spectrometer, with ESI-IT-MS configuration. Direct infusion of the sample solutions was carried out at a concentration of about 10 ppm in acetonitrile/water containing 0.1% v/v formic acid. The infusion flow was adjusted to 5.0 μL/min and the electrospray source was operated in positive mode, applying 4.5 kV to the electrospray capillary.

(A)

(B)

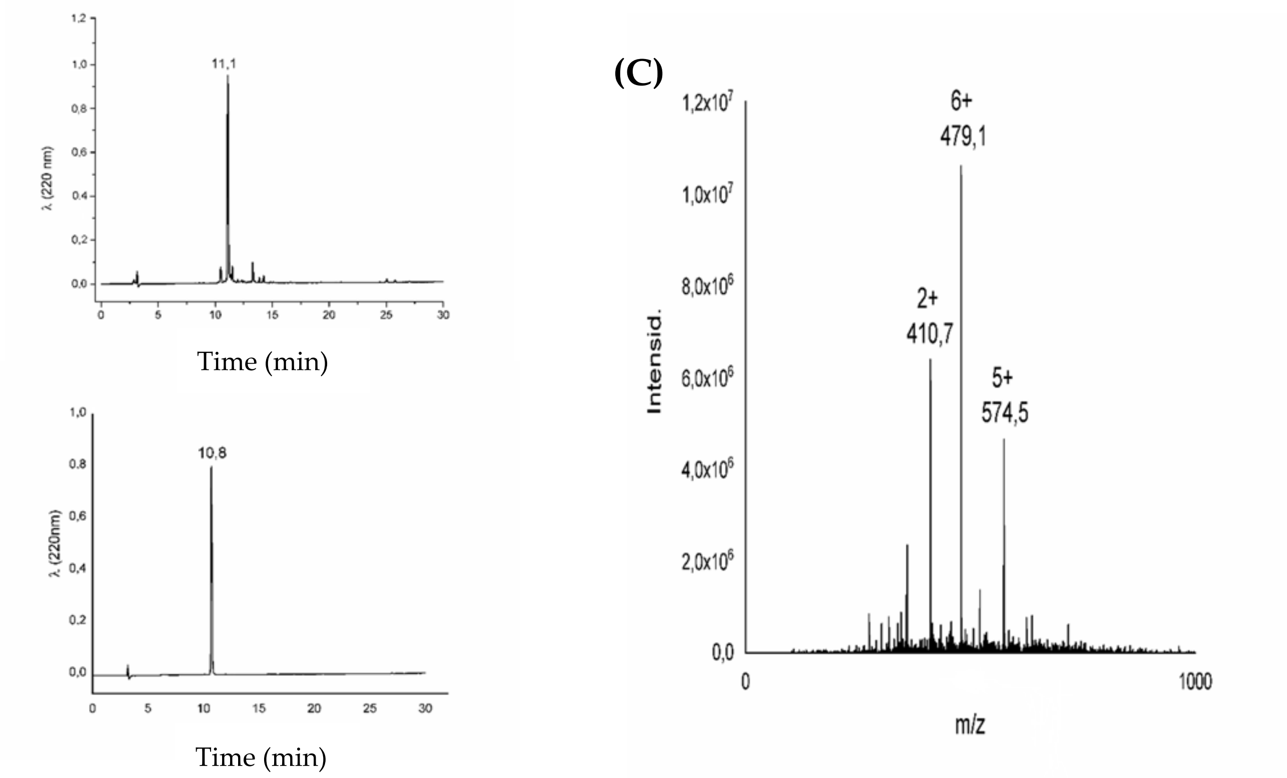

Figure S3. Chromatogram of the crude (a) and purified (KKYRYHLKPF)$_2$K (L-dimer) peptide (b) - analytical reverse phase Phenomenex Jupiter C18 column (150 x 4.6 mm), packed with spherical 5 mm particle and 300 Å pore size, using a linear gradient of 5–95% (v/v) of solvent B for 30 min, at a flow rate of 1.0 mL/min and UV detection at 220 nm. Mass spectrum of the peptide (c) - Thermo LCQ-fleet mass spectrometer, with ESI-IT-MS configuration. Direct infusion of the sample solutions was carried out at a concentration of about 10 ppm in acetonitrile/water containing 0.1% v/v formic acid. The infusion flow was adjusted to 5.0 μL/min and the electrospray source was operated in positive mode, applying 4.5 kV to the electrospray capillary.

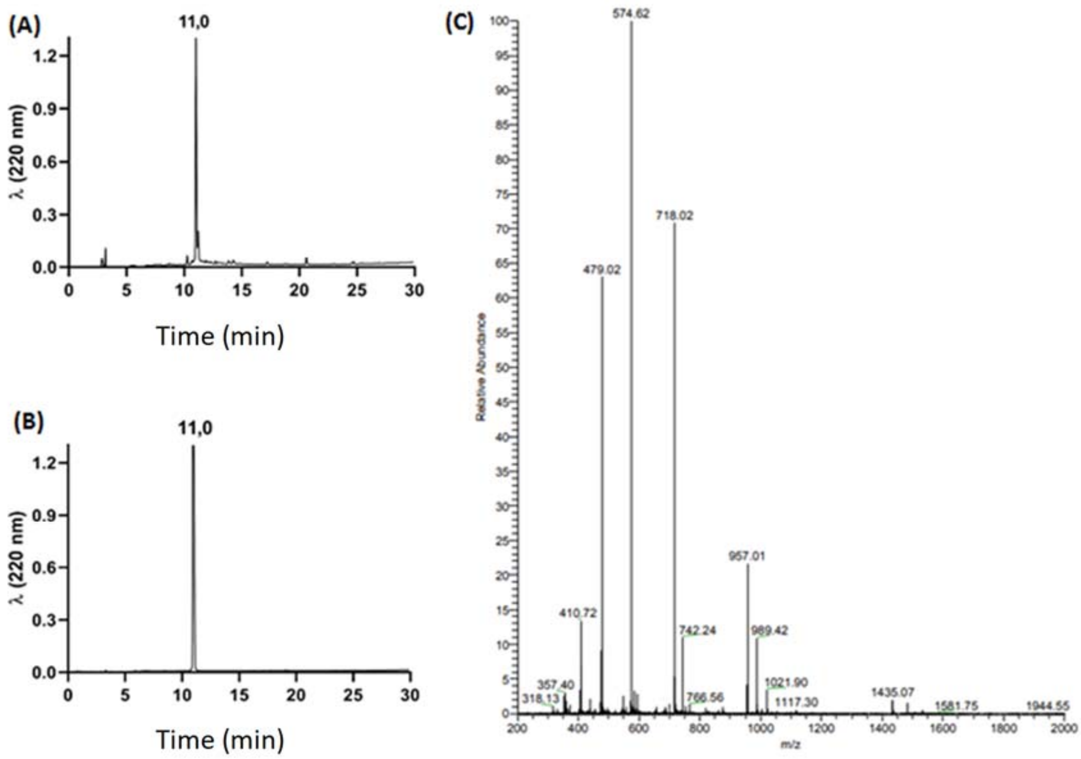

Figure S4. Chromatogram of the crude (a) and purified (kkyryhlkpf)₂K (D-dimer) peptide (b) - analytical reverse phase Phenomenex Jupiter C18 column (150 x 4.6 mm), packed with spherical 5 mm particle and 300 Å pore size, using a linear gradient of 5–95% (v/v) of solvent B for 30 min, at a flow rate of 1.0 mL/min and UV detection at 220 nm. Mass spectrum of the peptide (c) - Thermo LCQ-fleet mass spectrometer, with ESI-IT-MS configuration. Direct infusion of the sample solutions was carried out at a concentration of about 10 ppm in acetonitrile/water containing 0.1% v/v formic acid. The infusion flow was adjusted to 5.0 μL/min and the electrospray source was operated in positive mode, applying 4.5 kV to the electrospray capillary.

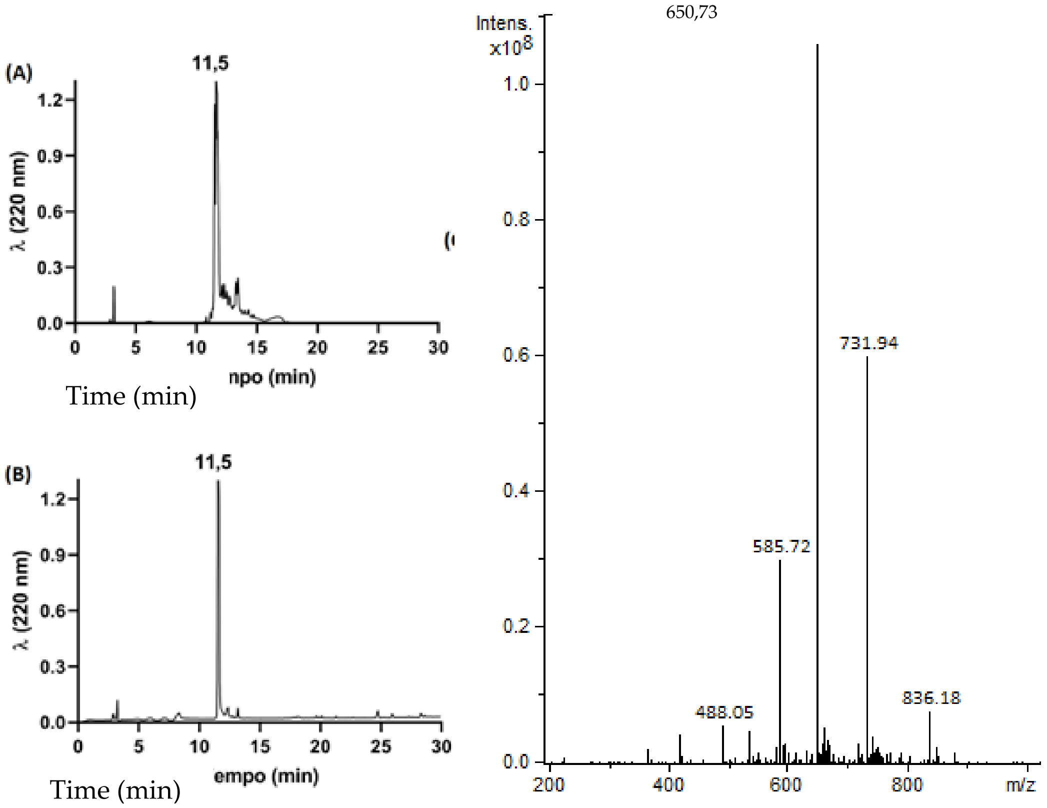

Figure S5. Chromatogram of the crude (a) and purified (KKYRYHLKPF)$_4$K$_2$K (L-tetramer) peptide (b) - analytical reverse phase Phenomenex Jupiter C18 column (150 x 4.6 mm), packed with spherical 5 mm particle and 300 Å pore size, using a linear gradient of 5–95% (v/v) of solvent B for 30 min, at a flow rate of 1.0 mL/min and UV detection at 220 nm. Mass spectrum of the peptide (c) - Thermo LCQ-fleet mass spectrometer, with ESI-IT-MS configuration. Direct infusion of the sample solutions was carried out at a concentration of about 10 ppm in acetonitrile/water containing 0.1% v/v formic acid. The infusion flow was adjusted to 5.0 μL/min and the electrospray source was operated in positive mode, applying 4.5 kV to the electrospray capillary.

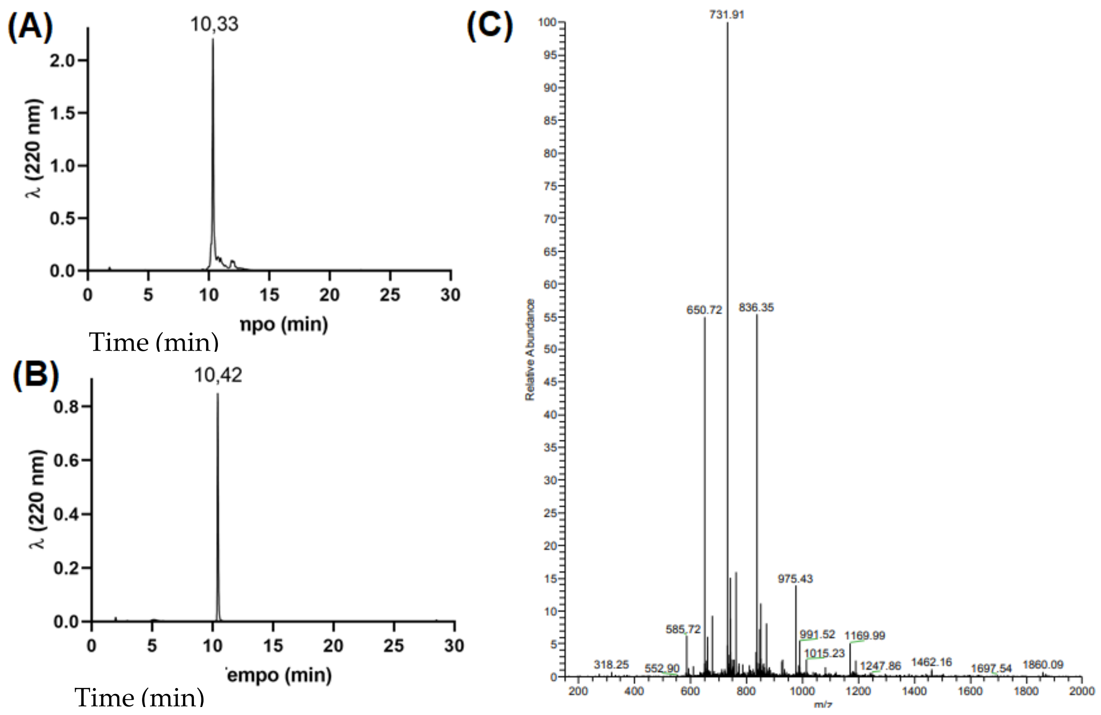

Figure S6. Chromatogram of the crude (a) and purified (kkyryhlkpf)$_4$K$_2$K (D-tetramer) peptide (b) - analytical reverse phase Phenomenex Jupiter C18 column (150 x 4.6 mm), packed with spherical 5 mm particle and 300 Å pore size, using a linear gradient of 5–95% (v/v) of solvent B for 30 min, at a flow rate of 1.0 mL/min and UV detection at 220 nm. Mass spectrum of the peptide (c) - Thermo LCQ-fleet mass spectrometer, with ESI-IT-MS configuration. Direct infusion of the sample solutions was carried out at a concentration of about 10 ppm in acetonitrile/water containing 0.1% v/v formic acid. The infusion flow was adjusted to 5.0 μL/min and the electrospray source was operated in positive mode, applying 4.5 kV to the electrospray capillary.

Table S1. Molecular weight and mass/charge ratio of the synthetic peptides.

**eptide**	**MW g/mol**	**mass/charge ratio**		
		**Peak 1**	**Peak 2**	**Peak 3**
L-monomer	1378.7	690.4/2	460.6/3	345.7/4
D-monomer	1378.7	690.4/2	460.6/3	345.7/4
L-dimer	2868.5	574.7/5	479.1/6	410.8/7
D-dimer	2868,5	718,1/4	574.7/5	479.1/6
L-tetramer	5848.1	732.0/8	650.8/9	585.8/10
D-tetramer	5848.1	836.4/7	732.0/8	650.8/9

PLpro Inhibition Assays

Our findings indicated that the analogs showed comparable inhibition profiles [2] and IC_{50} values. The inhibition profiles of L-dimer, D-dimer and L-tetramer was published

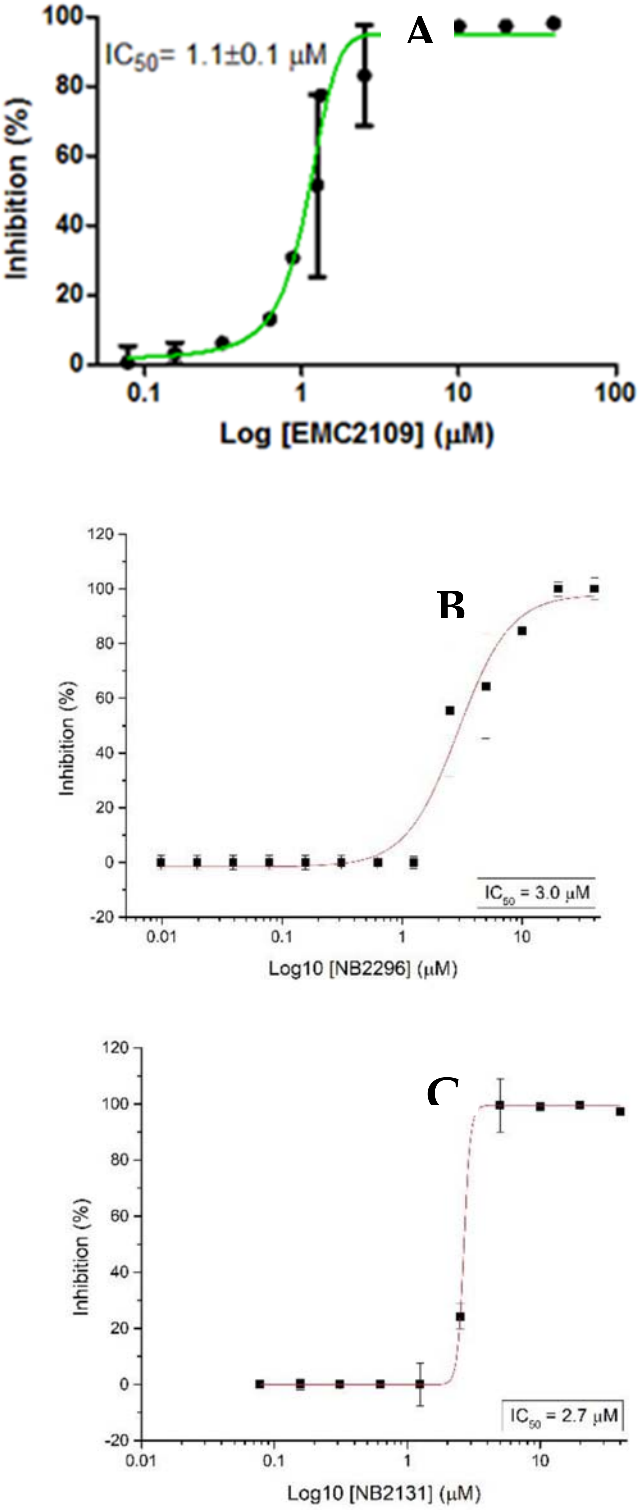

Figure S7. Representative concentration–response inhibition curves against PLpro from SARS-CoV-2 for L-monomer (A), D-monomer and D-tetramer. Two independent experiments were done.

1. Santos-Filho, N. A., Righetto, G. M., Pereira, M. R., Piccoli, J. P., Almeida, L. M. T., Leal, T. C., Camargo, I. L. B. C., Cilli, E. M. Effect of C-terminal and N-terminal dimerization and alanine scanning on antibacterial activity of the analogs of the peptide p-BthTX-I. *Peptide Science*, **2022**, *114:e24243*, 1-10. DOI: 10.1002/pep2.24243.
2. Freire, M. C.; Noske, G. D.; Bitencourt, N. V.; Sanches, P. R..; Santos-Filho, N. A.; Gawriljuk, V. O.; Souza E. P.; Nogueira, V. H. R.; Godoy, M. O.; Nakamura, A. M.; Fernandes, R. S.; Godoy, A. S.; Juliano, M. A.; Peres, B. M.; Barbosa, C. G.; Moraes, C. B.; Freitas-Junior, L. H. G.; Cilli, E. M.; Guido, R. V. C.; Oliva, G. Non-Toxic Dimeric Peptides Derived from the Bothropstoxin-I Are Potent SARS-CoV-2 and Papain-like Protease Inhibitors. *Molecules,* **2021**, *26(16*), 4896.
